# Gremlin Regulates Tubular Epithelial to Mesenchymal Transition via VEGFR2: Potential Role in Renal Fibrosis

**DOI:** 10.3389/fphar.2018.01195

**Published:** 2018-10-17

**Authors:** Laura Marquez-Exposito, Carolina Lavoz, Raul R. Rodrigues-Diez, Sandra Rayego-Mateos, Macarena Orejudo, Elena Cantero-Navarro, Alberto Ortiz, Jesús Egido, Rafael Selgas, Sergio Mezzano, Marta Ruiz-Ortega

**Affiliations:** ^1^Cellular Biology in Renal Diseases Laboratory, IIS-Fundación Jiménez Díaz, Universidad Autónoma de Madrid, Madrid, Spain; ^2^Red de Investigación Renal, Madrid, Spain; ^3^Division of Nephrology, School of Medicine, Universidad Austral, Valdivia, Chile; ^4^Laboratory of Nephrology, Fundación para la Investigación Biomédica del Hospital Universitario la Paz, Universidad Autónoma de Madrid, Madrid, Spain; ^5^Vascular and Renal Translational Research Group, Institut de Recerca Biomédica de Lleida, Lleida, Spain; ^6^Division of Nephrology and Hypertension, IIS-Fundación Jiménez Díaz, Universidad Autónoma de Madrid, Madrid, Spain; ^7^Spanish Biomedical Research Centre in Diabetes and Associated Metabolic Disorders, Madrid, Spain

**Keywords:** gremlin, VEGFR2, notch, EMT, tubular cells, fibrosis, renal

## Abstract

Chronic kidney disease (CKD) is emerging as an important health problem due to the increase number of CKD patients and the absence of an effective curative treatment. Gremlin has been proposed as a novel therapeutic target for renal inflammatory diseases, acting via Vascular Endothelial Growth Factor Receptor-2 (VEGFR2). Although many evidences suggest that Gremlin could regulate renal fibrosis, the receptor involved has not been yet clarified. Gremlin, as other TGF-β superfamily members, regulates tubular epithelial to mesenchymal transition (EMT) and, therefore, could contribute to renal fibrosis. In cultured tubular epithelial cells Gremlin binding to VEGFR2 is linked to proinflammatory responses. Now, we have found out that in these cells VEGFR2 is also involved in the profibrotic actions of Gremlin. VEGFR2 blockade by a pharmacological kinase inhibitor or gene silencing diminished Gremlin-mediated gene upregulation of profibrotic factors and restored changes in EMT-related genes. Moreover, VEGFR2 inhibition blocked EMT phenotypic changes and dampened the rate of wound healing in response to Gremlin. The role of VEGFR2 in experimental fibrosis was evaluated in experimental unilateral ureteral obstruction. VEFGR2 inhibition diminished the upregulation of profibrotic genes and EMT changes, as well as the accumulation of extracellular matrix proteins, such as fibronectin and collagens in the obstructed kidneys. Notch pathway activation participates in renal damage progression by regulating cell growth/proliferation, regeneration and inflammation. In cultured tubular epithelial cells, Notch inhibition markedly downregulated Gremlin-induced EMT changes and wound healing speed. These results show that Gremlin regulates the EMT process via VEGFR2 and Notch pathway activation, suggesting that the Gremlin/VEGFR2 axis could be a potential therapeutic target for CKD.

## Introduction

Chronic kidney disease (CKD) is a devastating disease that affects 5–7% of the worldwide population and is a strong forecaster of end-stage renal disease, cardiovascular morbidity, and mortality (Sanchez-Niño et al., 2017). The elevated incidence of obesity, diabetes and aging will greatly increase the number of CKD patients in a near future. Regardless of the underlying etiology, most renal diseases progress to permanent loss of kidney function caused by progressive and irreversible nephron loss and reduced regenerative capacity. CKD progression is characterized by sustained inflammation (Anders, 2014) and excessive accumulation of extracellular matrix (ECM) in the kidney, leading to tubulointerstitial fibrosis that is associated to renal function loss and end stage renal disease (Cortinovis et al., 2016). The current therapeutic armamentarium for CKD only slows disease progression, and novel therapeutic strategies are needed. Intensive research has focused on finding novel potential therapeutic targets for CKD, based on different approaches from anti-inflammatory treatments to novel epigenetic drugs (Duffield, 2014; Ruiz-Andres et al., 2016; Liu et al., 2018). A current hypothesis is that the re-emergence of developmental programs could participate in the pathogenesis of CKD and, therefore, their blockade could exert protective effects (Roxburgh et al., 2006). Among developmental genes, Gremlin has a potential relevance as a therapeutic target (Lappin et al., 2002; Mezzano et al., 2018).

Gremlin is a member of the DAN family of secreted Bone Morphogenetic Proteins (BMPs) antagonists, contains a cysteine-rich region and a cysteine knot motif responsible for BMP binding, whose structure is shared by members of the transforming growth factor beta (TGF-β) superfamily (Topol et al., 2000; Mezzano et al., 2018). Earlier studies demonstrate an important role of Gremlin in development, including nephrogenesis, acting as a BMP antagonist (Merino et al., 1999; Khokha et al., 2003; Michos et al., 2004). More recently, Gremlin has been suggested as an important promoter of fibrosis in different pathological conditions including renal, liver, lung, and myocardial diseases (Dolan et al., 2005; Carvajal et al., 2008; Mueller et al., 2013; Brazil, 2015; Erdmann et al., 2015; Mezzano et al., 2018). Moreover, *in vitro* studies have demonstrated direct effects of Gremlin in the regulation of profibrotic-related events (Zode et al., 2009; Li et al., 2012; Rodrigues-Diez et al., 2012; Huang et al., 2013). However, the potential Gremlin receptor involved in fibrotic processes has not been fully defined.

Renal fibrosis is a major hallmark of CKD, and finding an anti-fibrotic therapy is an unmet need. During the past decade, the origin of myofibroblasts, the primary source of ECM in scar tissue formation, has been intensively investigated. Current data strongly suggest that in the kidney these myofibroblasts may arise from a number of sources such as activation of tissue fibroblasts, migration of circulating mesenchymal progenitors or cell transitions, such as epithelial-to-mesenchymal transition (EMT) or endothelial-to-mesenchymal transition (EndoMT) (Zeisberg and Neilson, 2009; Duffield, 2014; Lovisa et al., 2015; Liu et al., 2018). Interestingly Gremlin can induce EMT of tubular epithelial cells and cancer cells (Li et al., 2012; Rodrigues-Diez et al., 2012; Rodrigues-Diez et al., 2014), and can activate other renal cells, including fibroblasts and mesangial cells to increase the production of ECM proteins, such as collagens (Rodrigues-Diez et al., 2012; Huang et al., 2013). However, the receptor involved in Gremlin-induced fibrosis and EMT has not been found out yet. Some studies suggest that Gremlin regulates fibrosis by its BMP antagonist activity (Myllärniemi et al., 2008; Staloch et al., 2015), whereas many other studies have observed cellular actions of Gremlin independently of BMP antagonism (Mezzano et al., 2018). Recently, the vascular endothelial growth factor receptor 2 (VEGFR2) has been described as a Gremlin receptor in endothelial and tubular epithelial cells, showing some differences to canonical ligands in binding affinity and downstream responses (Mitola et al., 2010; Corsini et al., 2014; Lavoz et al., 2015; Mezzano et al., 2018). We have recently described that Gremlin activates VEGFR2 signaling pathway in the murine kidney, mainly on tubular epithelial cells, and this is linked to the induction of an acute inflammatory response (Lavoz et al., 2015). Interestingly, activation of VEGFR2 signaling and re-expression of Gremlin in tubular epithelial cells has been observed in several human nephropathies (Lavoz et al., 2015), suggesting that the Gremlin/VEGFR2 axis could be involved in CKD progression.

Notch signaling is an evolutionarily conserved pathway involved in cell fate control during development, stem cell self-renewal and postnatal tissue differentiation (Siebel and Lendahl, 2017). This pathway is one of the most relevant mechanisms regulating EMT in many cell types, including carcinogenesis (Takebe et al., 2015). Levels of some Notch pathway components have been proposed as biomarkers of renal disease progression in human CKD and many preclinical studies have suggested that Notch inhibition could be a therapeutic option for renal diseases, by modulating, cell proliferation, inflammation and EMT (Bielesz et al., 2010; Murea et al., 2010; Sharma et al., 2011; Marquez-Exposito et al., 2018). We have previously described that Gremlin activates Notch signaling in the kidney leading into an acute inflammatory responses (Lavoz et al., 2018), however, the role of this pathway in Gremlin-induced EMT remains unstudied.

According to this background, we have investigated the potential role of VEGFR2 in the regulation of Gremlin-induced EMT in cultured tubular epithelial cells, and its role in renal fibrosis, testing the effects of VEGFR2 blockade in experimental renal fibrosis.

## Materials and Methods

### Ethics Statement

All animal procedures were performed according to the guidelines of animal research in the European Community and with prior approval by the Ethics Committee of the Health Research of the IIS-Fundación Jiménez Díaz.

### Experimental Model of Renal Fibrosis

The model of unilateral ureteral obstruction (UUO) was performed in male C57BL/6 mice under isoflurane-induced anesthesia. The left ureter was ligated with silk (5/0) at two locations and cut between ligatures to prevent urinary tract infection (obstructed kidney), as described (Lavoz et al., 2015) and mice were studied after 5 days. To examine the VEGFR2 pathway, some animals were treated daily with the VEGFR2 kinase inhibitor SU5416 (i.p; 0.1 mg per day, Vichem, Budapest, Hungary). To study the Notch pathway, some animals were treated daily with the Notch inhibitor DAPT (a γ-secretase inhibitor; i.p; 0.1 mg per day, Calbiochem). Both treatments were started 1 day before UUO surgery (*n* = 8 mice per group). At the time of sacrifice, animals were anesthetized with 5 mg/kg xylazine (Rompun, Bayer AG) and 35 mg/kg ketamine (Ketolar, Pfizer) and the kidneys were perfused *in situ* with cold saline before removal. A piece of the kidney (2/3) was fixed, embedded in paraffin, and used for immunohistochemistry, and the rest was snap-frozen in liquid nitrogen for renal cortex RNA and protein studies. In both models, studies compared the contralateral and obstructed kidney in each mouse. In addition, a control group of sham-operated mice showed the same results as contralateral kidneys (data not shown).

Paraffin-embedded kidney sections were stained using standard histology procedures. Immunostaining was carried out in 3 μm thick tissue sections. Renal fibrosis was evaluated by Sirius Red staining. Samples were mounted in non-aqueous medium DPX new (Merck-Millipore) and examined by a Nikon Eclipse E400 microscope. For quantification, the percentage stained area out of the total area was calculated in five randomly chosen fields (×200 magnification) using Image-Pro Plus software (Media Cybernetics, Washington) and results were expressed as fold-change over control.

### Cell Culture Studies

Human renal proximal tubular epithelial cells (HK2 cell line, ATCC CRL-2190) were grown in RPMI 1640 medium with 10% heat-inactivated fetal bovine serum (FBS), 2 mM glutamine, 100 U/ml penicillin, 100 μg/ml streptomycin, 5 μg/ml Insulin Transferrin Selenium (ITS) and 36 ng/ml hydrocortisone in 5% CO_2_ at 37°C. All the *in vitro* studies were done in HK2 cell line (limitation of the study). At confluence, cells were growth-arrested in serum-free medium for 24 h before the experiments. Cells were cultured in six-well plates and stimulated with vehicle or recombinant human Gremlin (PeproTech, 10 ng/mL) for 24 (gene expression) or 48 h (protein levels) in serum-free medium. In some experiments, cells were preincubated for 1 h with VEGFR2 kinase inhibitor SU5416 (5 μM; Vichem, Budapest, Hungary), or DAPT (30 nM, Calbiochem). DMSO, used as solvent, as well as SU5416 and DAPT alone had no effect on cell viability, gene and protein expression (data not shown). Cells were used for protein or RNA studies. Fibronectin was measured in supernatants (cell-conditioned media) and in total protein extract (cell-associated fraction).

### Cell Transfection and Gene Silencing

Gene silencing in cultured cells was performed using either a predesigned siRNA corresponding to the human KDR/VEGFR2 cDNA sequence (s7822; Ambion) or a non-specific control siRNA (Ambion). Subconfluent HK2 cells were transfected for 24 h with Lipofectamine^TM^ RNAiMAX Reagent (Invitrogen) according to the manufacturer’s guidelines. Then, cells were incubated in serum-free medium for 24 h before the experiments. Some cells were stimulated with Gremlin or vehicle for different time intervals.

### Wound Healing

For wound healing assays, a single scrape wound was made with a p20 pipette tip on a monolayer of cultured HK2 cells, and two pictures per well were taken at this time point (*t* = 0) using an inverted microscope (Leica DMI3000 B). Wounded cells were preincubated 1 h with treatments (SU5416: 5 μM or DAPT: 30 nM) and later stimulated with Gremlin (10 ng/mL) or TGF-β1 (1 ng/mL). Seventy-two hours later two pictures were taken again in the same point as at *t* = 0 and the gap area was measured using the Image-Pro Plus software (Media Cybernetics, Washington). Data are expressed as mean percentage of wound healing ± SEM.

### Protein Studies

Proteins were obtained from cells or mouse kidneys using lysis buffer (50 mmol/l Tris–HCl, 150 mol/l NaCl, 2 mmol/l EDTA, 2 mmol/l EGTA, 0.2% Triton X-100, 0.3% IGEPAL, 10 μl/ml proteinase inhibitor cocktail, 0.2 mmol/l PMSF, and 0.2 mmol/l orthovanadate). Protein levels were quantified using a Pierce^TM^ BCA protein assay kit (Thermo Scientific, Rockford, IL, United States). For Western blotting, cell protein extracts (20–25 μg/lane) or cell supernatants (25–30 μl/lane) were separated on 6–12% polyacrylamide-SDS gels under reducing conditions. Samples were then transferred onto PVDF membranes (Bio-Rad, Spain), blocked with TBS/5% non-fat milk/0.05% Tween-20, and incubated overnight at 4°C with the corresponding primary antibodies. After washing, membranes were incubated with the appropriate HRP (horseradish peroxidase)-conjugated secondary antibody (Amersham Biosciences) and developed using an ECL kit (Amersham Biosciences). The quality of proteins and efficacy of protein transfer were evaluated by Red Ponceau staining (data not shown). The loading control for soluble proteins (cell supernatants) was Red Ponceau staining and the albumin band (67 kDa) was used for quantification of the loading control. Digital chemiluminescence images were taken by LAS 4000 (GEHealthcare) and quantified by Quantity One^®^ software. The following primary antibodies were employed [dilution]: α-smooth muscle actin (α-SMA) ([1:1000], Sigma), E-cadherin ([1:500], CST), Fibronectin ([1:5000]; BD Pharmingen), panCytokeratin ([1:500], Sigma), Vimentin (E-5) ([1:1000]; Santa Cruz, sc-373717), α-tubulin ([1:5000]; Sigma) and GAPDH ([1:5000]; Chemicon International).

### Immunofluorescence Staining of EMT Markers

To determine EMT changes, specific EMT markers were assessed by immunofluorescence in control and Gremlin-stimulated cells for 72 h. Cells were fixed with 4% PFA, permeabilized with triton X-100 (0.2%), blocked with 4% BSA in PBS and incubated with the following primary antibodies [dilution]: Vimentin ([1:200], Santa Cruz, sc-373717), panCytokeratin (C11) ([1:200]; Santa Cruz, sc-8018) and α-SMA ([1:200], Sigma). Secondary antibodies were Alexa Fluor^®^-488 conjugated antibody ([1/200] A21206, Invitrogen) and Alexa Fluor^®^-633 conjugated antibody ([1/200]; A21206, Invitrogen). Nuclei were stained with 1 μg/ml DAPI (Sigma-Aldrich) as control of equal cell density. Absence of primary antibody was used as negative control. Samples were mounted in ProLong^TM^ Gold Antifade Reagent (Invitrogen by Thermo Fisher Scientific) and examined by a Leica TCS SP5 confocal microscope.

### Gene Expression Studies

RNA from cells or renal tissue (pulverized in a metallic chamber) was isolated with TriPure reagent (Roche). cDNA was synthesized by a High Capacity cDNA Archive kit (Applied Biosystems) using 2 μg of total RNA primed with random hexamer primers following the manufacturer’s instructions. Next, quantitative gene expression analysis was performed by real-time PCR on an AB7500 fast real-time PCR system (Applied Biosystems) using fluorogenic TaqMan MGB probes and primers designed by Assay-on-Demand^TM^ gene expression products. Human assays IDs were: *E-CADHERIN* (CDH1), Hs01023895_m1; *TFG-b* 1 (TGFB1), Hs00998133_m1; *CTGF*, Hs00170014_m1, *FIBRONECTIN*, Hs00401006_m1, and *VIMENTIN*: Hs00185584_m1. Mouse assays IDs were: *fibronectin* (Fn1), Mm01256744_m1; *type I collagen* (Col1a2), Mm00483888_m1; *tgf-β1* (tgfb1), Mm01178820_m1; *pai-1* (Serpine), Mm00435858_m1. Data were normalized to human *GAPDH* Hs02786624_g1, or mouse *gapdh*: Mm99999915_g1. The mRNA copy numbers were calculated for each sample by the instrument software using Ct value (“arithmetic fit point analysis for the lightcycler”). Results were expressed in copy numbers, calculated relative to unstimulated cells after normalization against GAPDH.

### Statistical Analysis

Results throughout the text are expressed as mean ± SEM of fold increase over control. Differences between groups were assessed by Student t (cells) and Mann-Whitney (mice) tests. Statistical significance was assumed when a null hypothesis could be rejected at *p* < 0.05. Statistical analysis was performed using the SPSS statistical software, version 16.0, Chicago, IL, United States.

## Results

### Gremlin via VEGFR2 Regulates the Expression of Many Genes Involved in Fibrosis in Cultured Tubular Epithelial Cells

**FIGURE 1:**
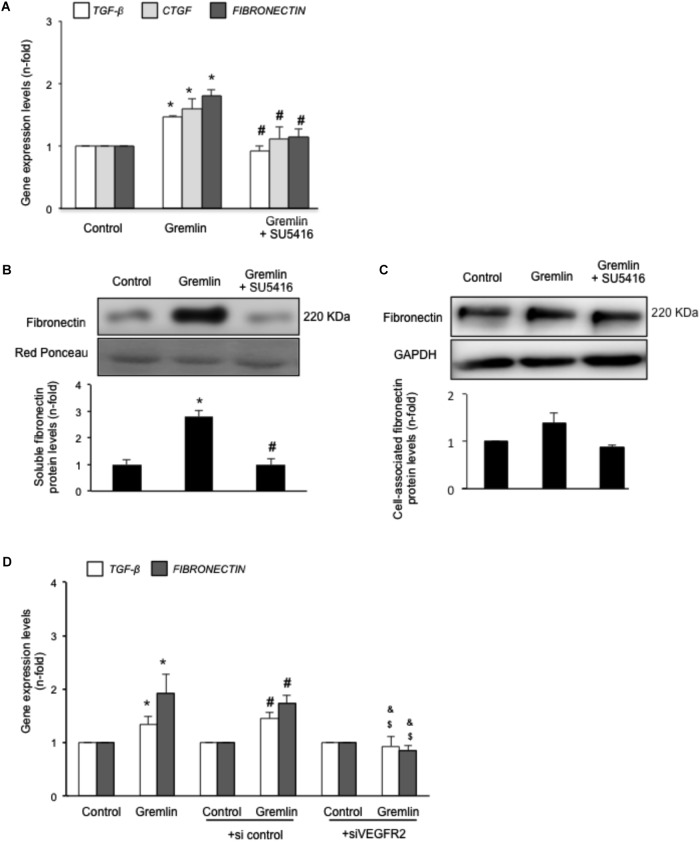
Gremlin via VEGFR2 regulates profibrotic factors and ECM components in cultured human tubular epithelial cells. Cells (HK2 cell line) were preincubated with the VEGFR2 kinase inhibitor SU5416 (5 μM) or vehicle before stimulation with Gremlin (10 ng/ml). **(A)** Gene expression was evaluated 24 h after Gremlin stimulation. Total cell RNA was isolated to assess mRNA levels of TGF-β, CTGF, and FIBRONECTIN by quantitative real-time PCR. **(B)** Effect of VEGFR2 blockade on Gremlin-induced ECM protein production was evaluated by quantifying soluble fibronectin levels by western blot, after 48 h of incubation. Figures show a representative experiment of soluble **(B)** and cell associated **(C)** Fibronectin (upper panel) and quantification (lower panel). Results are expressed as mean ± SEM of six independent experiments. ^∗^*p* < 0.05 vs. control, ^#^*p* < 0.05 vs. Gremlin. **(D)** VEGFR2 gene silencing blocked Gremlin-induced gene upregulation of matrix-related factors. HK2 cells were transfected with a siRNA against VEGFR2 or siRNA scrambled as described in Materials and Methods. Then, cells were stimulated or not with 10 ng/mL Gremlin for 24 h. Data are expressed as mean ± SEM of five independent experiments. ^∗^*p* < 0.05 vs. control untransfected; ^#^*p* < 0.05 vs. untreated control siRNA-transfected cells; ^$^*p* < 0.05 vs. Gremlin-treated control siRNA-transfected cells; ^&^*p* < 0.05 vs. Gremlin-treated untransfected cells.

Gremlin binds to VEGFR2 present in tubular epithelial cells *in vivo* and *in vitro* (Lavoz et al., 2015). Previous studies in cultured tubular epithelial cells have shown Gremlin regulates several profibrotic factors and ECM components (Li et al., 2012; Rodrigues-Diez et al., 2012, 2014), however, the role of VEGFR2 in these processes had not been investigated. Preincubation of cultured human tubular epithelial cells (HK2 cell line) with the pharmacological VEGFR2 kinase inhibitor SU5416 diminished Gremlin-mediated gene upregulation of profibrotic factors, including TGF-β1 and CTGF, and ECM components, such as fibronectin, evaluated by real time PCR (**Figure 1A**). Moreover, Gremlin-induced production of soluble fibronectin was blocked by treatment with the VEGFR2 kinase inhibitor, as determined by western blot (**Figure 1B**). Cell-associated fibronectin showed the same tendency but not statistical differences were found (**Figure 1C**).

The involvement of VEGFR2 on profibrotic gene regulation was further evaluated by gene silencing. HK2 cells were transfected with a siRNA against VEGFR2 or its corresponding scrambled control siRNA, and then stimulated with Gremlin for 24 h. In VEGFR2-silenced cells, Gremlin did not increase the gene expression of profibrotic factors and ECM related proteins, whereas in untransfected cells or in cells transfected with a control siRNA, Gremlin increased all those genes (**Figure 1D**).

### Gremlin Induces EMT in Cultured Tubular Epithelial Cells via VEGFR2 Signaling Activation

**FIGURE 2:**
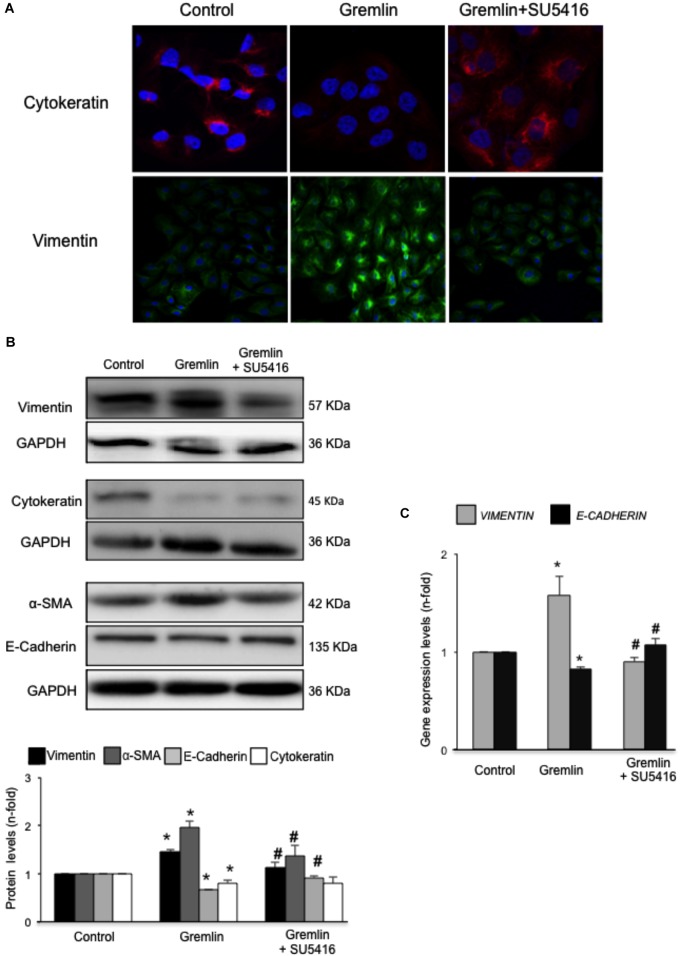
Gremlin via VEGFR2 induces EMT in tubular epithelial cells. **(A)** Cells (HK2 cell line) were preincubated with the VEGFR2 kinase inhibitor SU5416 (5 μM) or vehicle before stimulation with Gremlin (10 ng/ml) for 72 h. By confocal microscopy changes in epithelial phenotype and EMT-markers were evaluated. Vimentin is shown in green and Cytokeratin in red. Figure shows a representative experiment of two done by confocal microscopy. **(B)** Changes in EMT-related markers were quantified by western blot Gremlin diminished the levels of the epithelial markers E-Cadherin and Cytokeratin and increased the expression of mesenchymal markers, α-SMA and vimentin. All these changes were prevented by VEGFR2 inhibition. **(C)** Gene expression was evaluated 24 h after Gremlin stimulation. Total cell RNA was isolated to assess mRNA levels of VIMENTIN, and E-CADHERIN by quantitative real-time PCR. Figure shows mean ± SEM of six independent experiments. ^∗^*p* < 0.05 vs. control; ^#^*p* < 0.05 vs. Gremlin.

**FIGURE 3:**
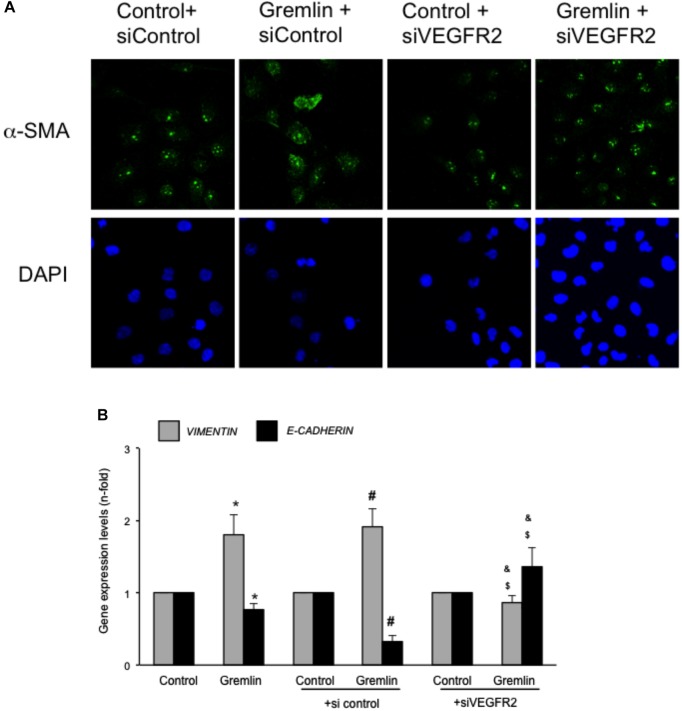
VEGFR2 gene silencing blocked Gremlin-induced EMT related changes in tubular epithelial cells. Sub-confluents HK2 cells were transfected with a siRNA against VEGFR2 or siRNA scrambled during 48 h. Then, cells were stimulated or not with 10 ng/mL Gremlin for 72 h. **(A)** Changes in the representative marker of activated myofibroblasts α-SMA was evaluated by confocal microscopy. To see cell density the nuclear marker DAPI was used. Figure shows a representative experiment of two done by confocal microscopy. **(B)** Gene expression was evaluated in silenced cells 24 h after Gremlin stimulation. Total cell RNA was isolated to assess mRNA levels of VIMENTIN, and E-CADHERIN by quantitative real-time PCR. Data are expressed as mean ± SEM of five independent experiments. ^∗^*p* < 0.05 vs. control untransfected; ^#^*p* < 0.05 vs. untreated control siRNA-transfected cells; ^$^*p* < 0.05 vs. Gremlin-treated control siRNA-transfected cells; ^&^*p* < 0.05 vs. Gremlin-treated untransfected cells.

An early study observed that during the transdifferentiation of tubular epithelial cells to fibroblasts, one of the most upregulated genes was Gremlin (Murphy et al., 2002). Latter, we described that Gremlin, both by stimulation with a recombinant Gremlin protein or by transfection with a Gremlin expression vector, could induce EMT in cultured human tubular epithelial cells (Rodrigues-Diez et al., 2012, 2014). Now, we have investigated whether VEGFR2 behaves as a Gremlin receptor involved in EMT regulation. EMT is initiated by the disruption of intercellular junctions resulting in a loss of their apical-basolateral polarity, and the acquisition of a front-back polarity. In HK2 cells, stimulation with Gremlin (10 ng/ml) decreased the expression of proteins that keep basolateral polarity, such as the epithelial marker Cytokeratin, as shown by confocal microscopy, that was restored in cells pretreated with the VEGFR2 inhibitor SU5416 (**Figure 2A**). EMT was also characterized by *de novo* synthesis of mesenchymal markers, such as α-SMA and vimentin (Zeisberg and Neilson, 2009). Gremlin induced vimentin expression associated to a change in cell phenotype from the typical cobblestone pattern of an epithelial monolayer to myofibroblast morphology, which was prevented by SU5416 (**Figure 2A**). Changes in EMT-related proteins were also confirmed by western blot. Gremlin decreased the protein levels of Cytokeratin and E-cadherin, essential proteins for the structural integrity of the renal epithelium, and increased vimentin and α-SMA levels, which were prevented by VEGFR2 inhibition in the case of e-cadherin, a-SMA and Vimentin (**Figure 2B**). To further demonstrate the role of VEGFR2 in the regulation of Gremlin-induced EMT process, gene-silencing experiments were done. The evaluation of changes in the representative marker of activated myofibroblasts α-SMA by confocal microscopy clearly showed that stimulation with Gremlin in VEGFR2 gene silenced cells, was not able to induce the phenotype changes and there was lower staining intensity compared to Gremlin-treated control siRNA transfected cells (**Figure 3A**). Additionally, SU5416 pretreatment or VEGFR2 gene silencing also modulated Gremlin-induced changes at the gene expression level, as observed for E-Cadherin and vimentin mRNA levels (**Figures 2C**, **3B**).

### Gremlin via VEGFR2 Modulates Wound Healing in Cultured Tubular Epithelial Cells

**FIGURE 4:**
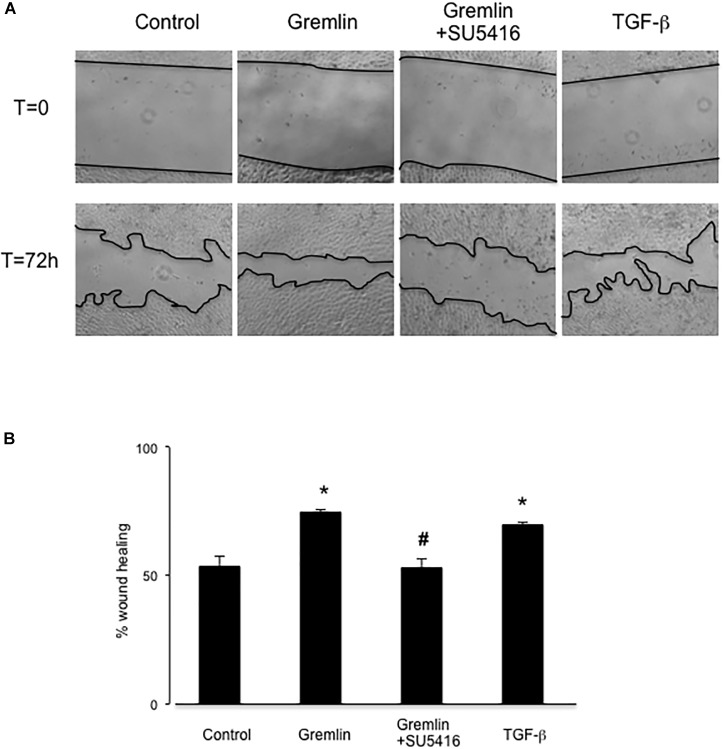
Gremlin via VEGFR2 regulates cells migration and proliferation in wound healing experiments. A monolayer of epithelial cells (HK2 cell line) was used as starting point. A single scrape wound was made in all cells and then they were stimulated with Gremlin (10 ng/ml) in the presence or absence of VEGFR2 kinase inhibitor SU5416 (5 μM, preincubated 1 h before). After 72 h, cells were evaluated under inverted microscopy. **(A)** Representative images of wound healing experiments and **(B)** their quantification. Results are expressed as mean ± SEM of four independent experiments, done by duplicate. ^∗^*p* < 0.05 vs. control; ^#^*p* < 0.05 vs. Gremlin.

Wound healing assays showed that Gremlin (10 ng/ml) increased the speed of epithelial cells invading the scar region compared to untreated cells, evaluated after 72 h (**Figure 4**). This response was similar to that observed in response to TGF-β1 stimulation (10 ng/ml), used as a positive control. The VEGFR2 kinase inhibitor SU5416 significantly diminished the percentage of wound healing induced by Gremlin (**Figure 4**).

### VEGFR2 Blockade Inhibits Experimental Renal Fibrosis

**FIGURE 5:**
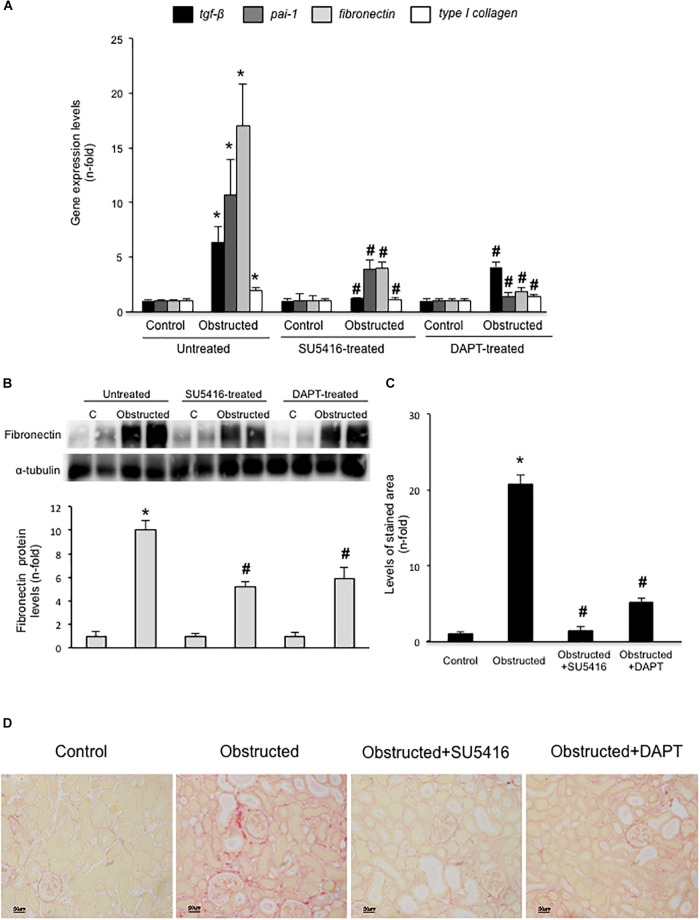
VEGFR2 kinase inhibition ameliorates experimental renal fibrosis in the model of unilateral ureteral obstruction. Mice were treated with SU5416 (i.p; 0.1 mg per day) starting 24 h before UUO surgery and were studied after 5 days. To evaluate the role of the Notch pathway, some mice were also treated with the Notch inhibitor DAPT (i.p; 0.1 mg per day). **(A)** RNA was obtained from total renal extracts and *tgf-b1, pai-1, fibronectin*, and *type I collagen* mRNA levels were determined by RT-qPCR. **(B)** Fibronectin protein levels were evaluated in total renal extracts by Western blotting. Levels of α-tubulin were used as a loading control. Figure shows several representative mice from each group and the quantification of the Western blot data. **(C,D)** Collagen deposition was evaluated in paraffin-embedded sections by Sirius Red staining, quantification assessed the stained area as a proportion of total area. **(D)** Figures show a representative mouse from each group. Magnification 200×, and **(C)** the quantification. Data are expressed as mean ± SEM of 6–8 animals per group. ^∗^*p* < 0.05 vs. control; ^#^*p* < 0.05 vs. UUO 5 days.

Gremlin administration in the murine kidney induces an acute inflammatory response, however, there was no increased ECM deposition in the kidney (Lavoz et al., 2018). Therefore, to evaluate the potential *in vivo* effects of Gremlin on renal fibrosis the UUO model was employed. This model is characterized by tubulo-interstitial fibrosis, observed as early as 5 days followed renal injury (Ucero et al., 2014). Moreover, in obstructed kidneys, Gremlin is increased and is associated to VEGFR2 activation (Lavoz et al., 2015). VEGFR2 kinase inhibition in obstructed kidneys prevented the increase in the expression of profibrotic (TGF-β1 and PAI-1) and matrix-related (fibronectin and type I collagen) genes that was observed in untreated obstructed kidneys (**Figure 5A**) and prevented the increase in Fibronectin protein (**Figure 5B**). The effect of VEGFR2 inhibition on renal fibrosis was further evaluated by Sirius red staining (**Figure 5D**). This technique disclosed a clear diminution of collagen deposition in mice treated with VEGFR2 inhibition as compared to untreated obstructed kidneys (**Figure 5C**). These data show that inhibition of VEGFR2 signaling ameliorates renal fibrosis and suggest that blockade of Gremlin/VEGFR2 could be responsible for downregulation of profibrotic events in injured kidneys.

### Blockade of Notch Pathway Activation Inhibited Experimental Renal Fibrosis

In some experimental models, Notch blockade ameliorates renal damage, mainly by inhibiting fibroblast proliferation and therefore, decreasing fibrosis (Bielesz et al., 2010; Marquez-Exposito et al., 2018). Treatment of obstructed mice with the γ-secretase inhibitor DAPT, an inhibitor of Notch activation, prevented the increase in renal profibrotic gene expression (**Figure 5A**), fibronectin levels (**Figure 5B**), and collagen deposition (**Figures 5C,D**) to levels similar to contralateral kidneys. These data confirms and extend previous observations (Bielesz et al., 2010).

### Notch Pathway Activation Is Involved in Gremlin-Induced EMT

**FIGURE 6:**
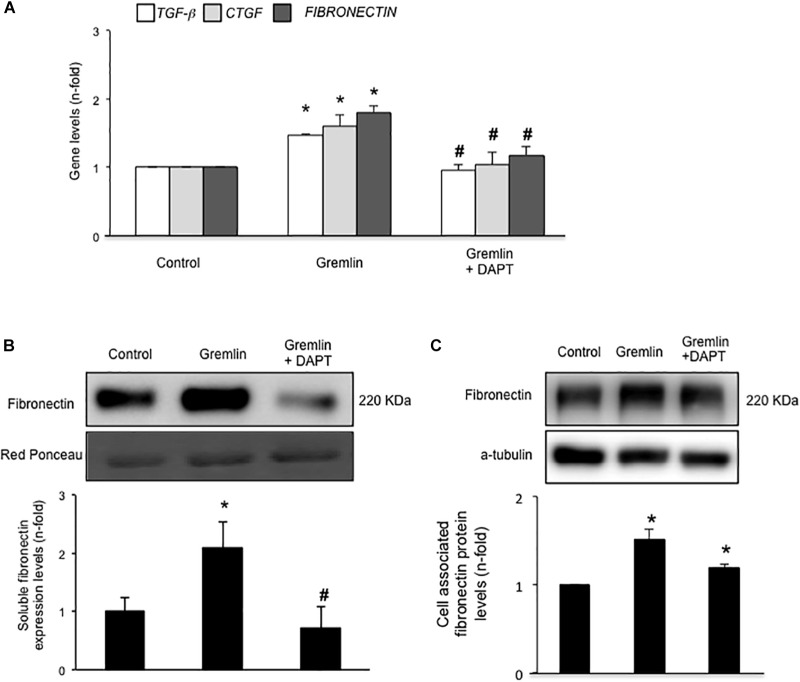
Gremlin via Notch pathway regulates profibrotic factors and ECM components in cultured human tubular epithelial cells. Cells (HK2 cell line) were preincubated with DAPT (30 nM) before stimulation with Gremlin (10 ng/ml). **(A)** Gene expression levels were evaluated 24 h after Gremlin stimulation. Total cell RNA was isolated to assess mRNA levels by RT-qPCR. Fibronectin levels were evaluated after 48 h in cell supernatants **(B)** and in total protein extracts **(C)**. A representative western blot experiment is shown in upper panel and quantification in the lower panel. ^∗^*p* < 0.05 vs. control, ^#^*p* < 0.05 vs. Gremlin.

**FIGURE 7:**
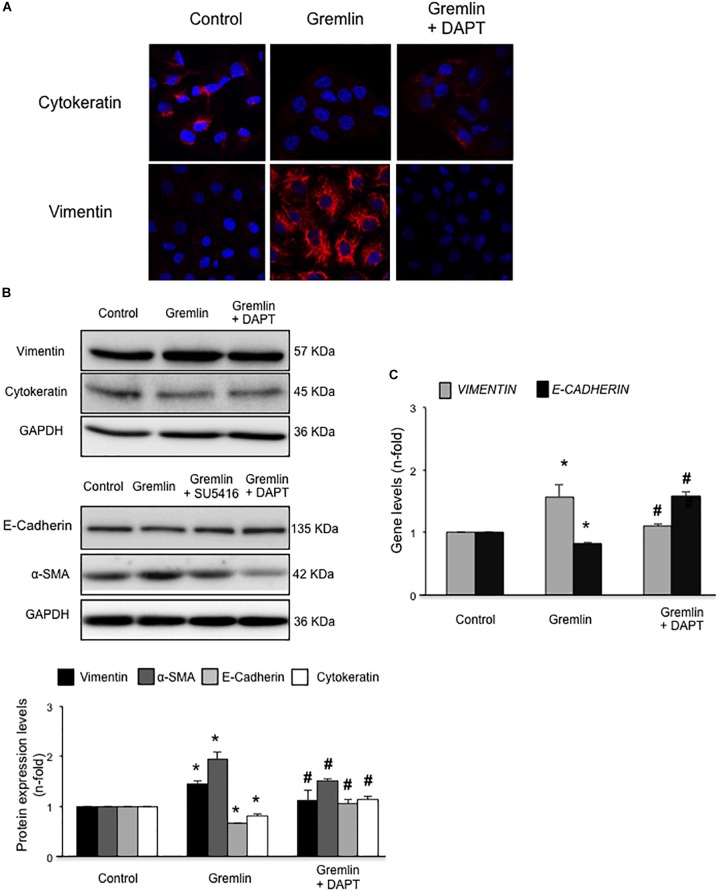
Gremlin via Notch pathway induces EMT in cultured human tubular epithelial cells. Cells (HK2 cell line) were preincubated with DAPT (30 nM) before stimulation with Gremlin (10 ng/ml). Changes in EMT-related markers were assessed by confocal microscopy **(A)** or western blot **(B)**. *Please, note that the pictures of e-cadherin, α-sma and their corresponding loading control (GAPDH) are the same images as shown in the* **Figure 2B** *but, in this case, Gremlin + DAPT point is included in the fourth lane*. Results are expressed as mean ± SEM of five independent experiments. All changes induced by Gremlin were prevented by Notch inhibition. Figure **(A)** shows a representative experiment out of two performed by confocal microscopy and **(B)** shows representative images of western blot and quantification. **(C)** Gene expression levels were evaluated 24 h after Gremlin stimulation. Total cell RNA was isolated to assess mRNA levels by RT-qPCR. Data are expressed as mean ± SEM of six independent experiments. ^∗^*p* < 0.05 vs. control; ^#^*p* < 0.05 vs. Gremlin.

**FIGURE 8:**
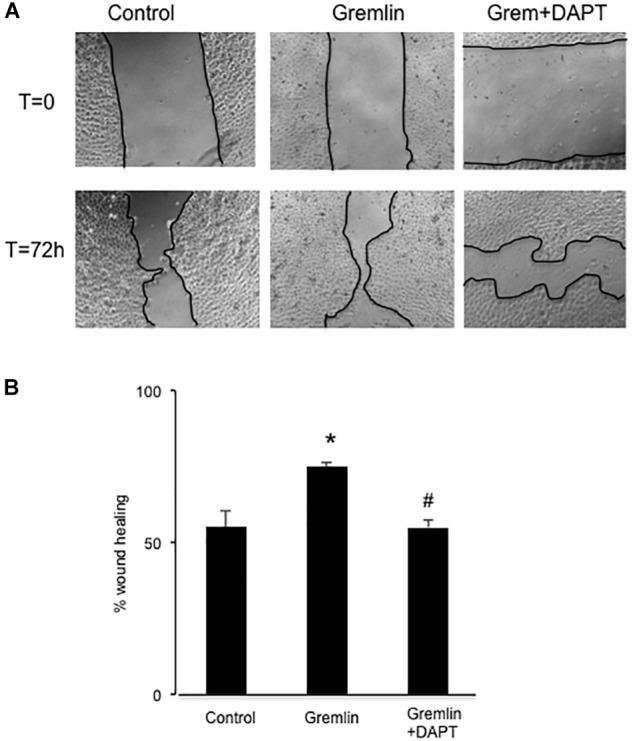
Effect of Notch inhibition on Gremlin-induced changes in wound healing assays. A monolayer of epithelial cells (HK2 cell line) was used as starting point. A single scrape wound was made. Then cells were preincubated with DAPT (30 nM) or vehicle before stimulation with Gremlin (10 ng/ml). After 72 h, cells were evaluated under an inverted microscopy. **(A)** Representative images of wound healing experiments and **(B)** their quantification. Results are expressed as mean ± SEM of four independent experiments, performed by duplicate. ^∗^*p* < 0.05 vs. control; ^#^*p* < 0.05 vs. Gremlin.

Finally, we further investigated whether the Notch pathway could be directly involved in Gremlin profibrotic responses. Preincubation with the γ-secretase inhibitor DAPT diminished Gremlin-induced changes in profibrotic gene expression and ECM components (**Figure 6**). Changes caused by Gremlin in the cell morphology and in EMT-related genes and proteins levels were prevented by DAPT (**Figure 7**). Moreover, the effects of Gremlin on wound healing speed were also inhibited by DAPT (**Figure 8**). DAPT alone did not show effect on wound healing when compared to control (Pazos et al., 2017).

## Discussion

In this paper we have described that in cultured tubular epithelial cells, VEGFR2 is the receptor involved in the regulation of the EMT program and of profibrotic-related genes in response to Gremlin. In human progressive CKD, Gremlin upregulation was associated to activation of VEGFR2 signaling, mainly in tubular epithelial cells (Lavoz et al., 2015). Moreover, Gremlin overexpression was found in areas of increased ECM deposition (Dolan et al., 2005; Mezzano et al., 2018), suggesting that this factor can participate in renal fibrosis. Our *in vivo* studies show anti-fibrotic effects of VEGFR2 inhibition in experimental kidney fibrosis, suggesting that the Gremlin/VEGFR2 axis could be a potential therapeutic target in renal disease.

In the kidney, Gremlin binds to VEGFR2 in tubular epithelial cells activating this receptor and subsequent downstream signaling pathways linked to regulation of renal inflammation (Lavoz et al., 2015, 2018). Now, our *in vitro* data in cultured tubular epithelial cells show that Gremlin via VEGFR2 activation regulates several gene programs to control profibrotic, ECM and EMT related factors. In other cell types Gremlin actions are also mediated by VEGFR2. In endothelial cells, Gremlin binds to VEGFR2 and activates this pathway regulating cell proliferation, migration, and angiogenesis (Shibuya and Claesson-Welsh, 2006; Mitola et al., 2010; Corsini et al., 2014). In skin keratinocytes and fibroblasts, Gremlin via VEGFR2 activates NF-E2-related factor 2 (NERF2) signaling and cell growth (Ji et al., 2016), and in retinal epithelial cells via mTOR regulates proliferation and migration (Liu et al., 2017). However, the receptor involved in Gremlin responses in other renal cells had not been investigated. In this sense, podocytes seem not to express VEGFR2 (Li et al., 2013). Our *in vitro* data suggest that VEGFR2 could be the receptor involved in Gremlin-mediated fibrotic responses in the kidney, but future research is needed in other kidney cell types beyond tubular cells.

Epithelial to mesenchymal transition is a cell reprogramming process in which epithelial cells acquire a mesenchymal phenotype. The contribution of tubular EMT to renal fibrosis is a matter of intense debate (LeBleu et al., 2013; Duffield, 2014; Lovisa et al., 2015; Liu et al., 2018). EMT is characterized by the loss of the epithelial properties of tubular epithelial cells, including permeability and polarity, which may result in decreased viability and contribute to renal injury (Duffield, 2014; Lovisa et al., 2015; Liu et al., 2018). Although in experimental and human CKD the number of myofibroblasts of tubular origin could be scarce, and it is not relevant to the total increase in collagen deposition, partial EMT is an initial step in renal damage and an important potential therapeutic target. In cultured tubular epithelial cells, we have found that VEGFR2 blockade, by the pharmacological inhibitor SU5416 or by gene silencing, inhibited the EMT program induced by Gremlin Therefore, Gremlin/VEGFR2 blockade could exert protective effects in renal injury by restoring or preventing the loss of epithelial integrity.

Epithelial to mesenchymal transition also participates in wound healing, malignant transformation and embryogenesis. In our study, we show that VEGFR2 is involved in Gremlin-induced wound healing in tubular epithelial cells. Gremlin induces EMT changes in other cell types, including airway epithelial cells (McCormack et al., 2013). In cancer cell lines, Gremlin besides EMT, also causes cell migration, invasion, and proliferation through BMP and VEGFR2-independent mechanisms (Kim et al., 2012; Yin et al., 2017; Mezzano et al., 2018). Future studies are needed to define the receptor involved in Gremlin-induced EMT in proliferative disorders.

Earliest studies showed that Gremlin could acts as a BMPs antagonist, mainly involved in embryogenesis, including kidney development (Merino et al., 1999; Khokha et al., 2003; Michos et al., 2004). However, the role of Gremlin as BMPs antagonists in other physiological and pathological conditions is controversial. Gremlin-induced inflammatory responses are BMP-independent, as described in endothelial and tubular epithelial cells, and are linked to activation of several transcription factors, NF-κB and Notch-1 (Corsini et al., 2014; Lavoz et al., 2015, 2018). Gremlin affinity for BMPs can vary between cells and pathological conditions, explaining the divergent results published. In isolated pancreatic stellate cells Gremlin acting as a BMP antagonist abolished BMP2’s suppression effects on TGF-β-induced collagen expression, by its binding to BMP2 and subsequent blockade of Smad1/5 phosphorylation, and therefore inhibiting BMP2 anti-fibrotic effects (Staloch et al., 2015). BMP7 have proven to be capable of reversing renal fibrosis in mice, EMT and *in vitro* profibrotic effects of TGF-β and Gremlin (Zeisberg et al., 2003; Weiskirchen and Meurer, 2013). In this regard, there is a complex relationship between BMPs and TGF-β signaling in the regulation of homeostasis and tissue fibrosis. Novel data indicate that Gremlin is another important player in this context. In cultured renal cells Gremlin is a downstream mediator of TGF-β profibrotic responses, including EMT of tubular epithelial cells (Rodrigues-Diez et al., 2012). *In vitro* Gremlin activates TGF-β/Smad pathway in tubular epithelial cells and in podocytes (Li et al., 2013; Rodrigues-Diez et al., 2014). Additionally, TGF-β blockade inhibited Gremlin-induced EMT (Rodrigues-Diez et al., 2014). All these data show a close interrelation between TGF-β and Gremlin, acting both as profibrotic factors.

Many experimental evidences suggest that Gremlin could be an important promoter of fibrosis in different pathologies, including liver fibrosis, lung diseases (particularly pulmonary hypertension and idiopathic pulmonary fibrosis), myocardial fibrosis, and chronic pancreatitis (Khokha et al., 2003; Mueller et al., 2013; Brazil, 2015; Mezzano et al., 2018). In several human renal diseases, Gremlin was found overexpressed, mainly in areas of tubulointerstitial fibrosis (Murphy et al., 2002; Dolan et al., 2005; Carvajal et al., 2008), suggesting that Gremlin could be involved in the fibrotic process during CKD. Several studies have demonstrated a direct fibrogenic effect of Gremlin in renal cells. In mesangial cells, Gremlin increased cell proliferation and ECM accumulation via ERK (Huang et al., 2013) and in renal fibroblasts Gremlin increased ECM production (Rodrigues-Diez et al., 2012). Accordingly, we have recently observed that in a model of Gremlin-induced renal damage there was an upregulation of fibrotic related genes, including TGF-β1, which were blocked by VEGFR2 kinase inhibition (Lavoz et al., 2018). However, no renal fibrosis was observed. Now, we have found out that experimental renal fibrosis was prevented by VEGFR2 blockade. In the UUO model, treatment with a VEGFR2 kinase inhibitor significantly prevented the renal overexpression of profibrotic factors and accumulation of key ECM proteins such as collagen. Interestingly, blocking VEGFR2 by circulating soluble receptor ectodomains, diminished fibrosis and capillary rarefaction in UUO (Lin et al., 2011). In that study, VEGFR2 blockade also blocked pericyte PDGFRβ activation, and pericyte differentiation and proliferation (Lin et al., 2011), thus blocking another source of scar-producing myofibroblasts. Unfortunately, that study did not assess Gremlin levels nor the role of Gremlin/VEGFR2 responses on endothelial cells. In mice, Gremlin blockade diminished ECM accumulation, as observed in streptozotocin-induced diabetes in knockout mice heterozygous for Grem1 (Roxburgh et al., 2009) and by Gremlin gene silencing (Zhang et al., 2010). Moreover, transgenic mice overexpressing Grem-1 specifically in tubular cells presented increased susceptibility to renal damage, induced by streptozotocin or folic acid administration (Droguett et al., 2014; Marchant et al., 2015). Accordingly, specific deletion of Grem-1 in tubular cells diminished renal fibrosis in folic acid-induced damage in mice (Church et al., 2017). These data show that inhibition of VEGFR2 signaling ameliorates renal fibrosis and suggest that Gremlin/VEGFR2 blockade could be responsible of the downregulation of profibrotic events, suggesting a novel anti-fibrotic therapy for CKD.

Activation of the Notch pathway participates in renal damage progression, but the precise mechanisms are not fully elucidated (Marquez-Exposito et al., 2018). In some experimental models of renal damage, including acute kidney injury models, such as folic acid administration in mice, Notch blockade by pharmacological inhibitors or soluble Notch ligands ameliorates renal damage, mainly by inhibiting fibroblast proliferation and therefore decreasing fibrosis (Bielesz et al., 2010; Murea et al., 2010; Sharma et al., 2011; Marquez-Exposito et al., 2018). More recently, anti-inflammatory properties of Notch inhibition have been described in experimental renal damage (Lavoz et al., 2018). In a previous study in the UUO model, authors demonstrated that a γ-secretase inhibitor downregulated gene expression of fibronectin and type I Collagen (Bielesz et al., 2010). Here, we have extended these data at the protein level showing that DAPT prevented the increase of profibrotic factors, including TGF-β1, and the renal accumulation of collagen and fibronectin in a UUO model. Notch is involved in EMT in carcinogenesis (Takebe et al., 2015; De Francesco et al., 2018). In cultured tubular epithelial cells, we found that DAPT diminished Gremlin-induced EMT changes, confirming the role of the Notch pathway in EMT regulation.

Chronic progressive kidney fibrosis remains an unresolved challenge. Irrespective of the underlying cause, CKD is linked to the development of tubulo-interstitial fibrosis. Our data show that blockade of Gremlin-mediated VEGFR2/Notch activation ameliorates fibrotic events and EMT processes, suggesting that Gremlin might be a fibrosis driver through VEGFR2 and Notch pathway, and a possible therapeutic target.

## Author Contributions

LM-E and CL contributed to the design of the experiments, acquisition, analysis, and interpretation of all data, and drafted the manuscript. RR-D contributed to design of the experiments, analysis and interpretation of data, and drafted the manuscript. SR-M, MO, and EC-N participated in the development of mouse models and analysis of data. AO, SM, RS, and JE contributed to the critical review of the manuscript and the financial support of the work. MR-O contributed to the design of the experiments, analysis and interpretation of the all data, draft of the manuscript, and financial support of the experiments. All authors have reviewed the manuscript and approved the final version.

## Conflict of Interest Statement

The authors declare that the research was conducted in the absence of any commercial or financial relationships that could be construed as a potential conflict of interest.
